# Supplementary material for: Overexpression of Bacterial Beta-Ketothiolase Improves Flax (Linum usitatissimum L.) Retting and Changes the Fibre Properties
Source: Metabolites. 2023 Mar 17;13(3):437. doi: 10.3390/metabo13030437 (PMC10052753; doi:10.3390/metabo13030437)
Supplement: Supplementary file 1 [file metabolites-13-00437-s001.zip › Table S1.pdf]

**GENE**	**5'-***Forward primer***-3'**	**5'-***Reverse primer***-3'**
Primers for the reference gene		
ACTIN	CCGGTGTTATGGTTGGAAT	TGTAGAAAGTGTGATGCCAAA
Primers amplifying the gene fragment encoding endogenous beta-ketothiolase		
Lu_bKAT	TTGCTTCCCAATTTGTCTACT	ATTGAGATGACTCCGAAACG
Primers amplifying the gene fragment encoding bacterial beta-ketothiolase		
phbA	CCGGCAAGTTTGACGAAGAG	GGCGTTGGCATAGCTCTTGA

Table S1
