# Supplementary figures and images for: Overexpression of Bacterial Beta-Ketothiolase Improves Flax (Linum usitatissimum L.) Retting and Changes the Fibre Properties
Source: Metabolites. 2023 Mar 17;13(3):437. doi: 10.3390/metabo13030437 (PMC10052753; doi:10.3390/metabo13030437)

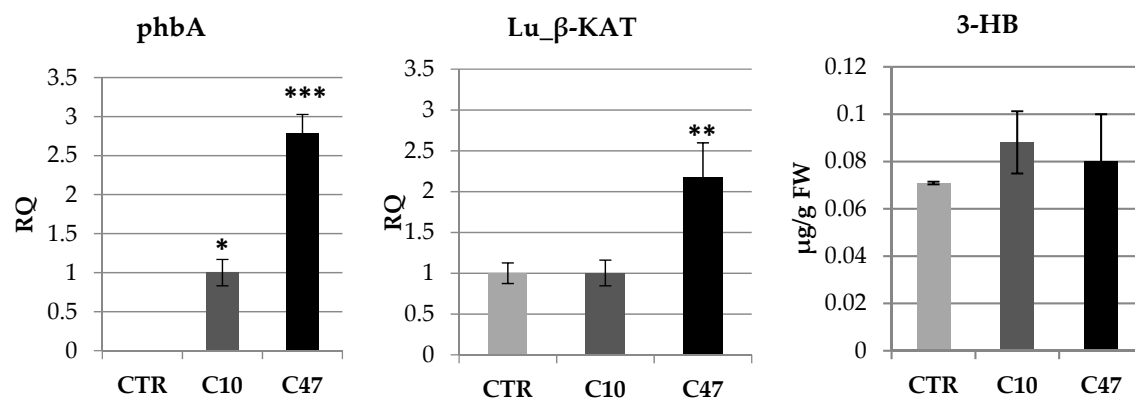

**Figure S1**

Supplement: Supplementary file 1 [file metabolites-13-00437-s001.zip › Figure S1.pdf]

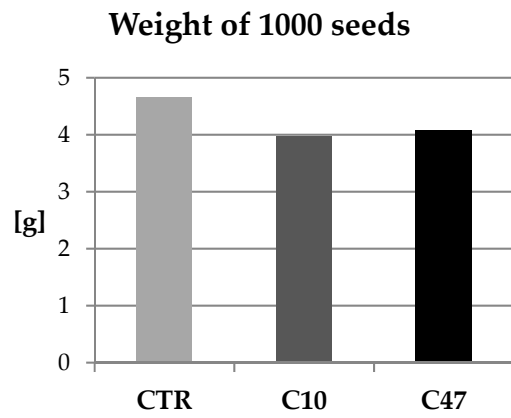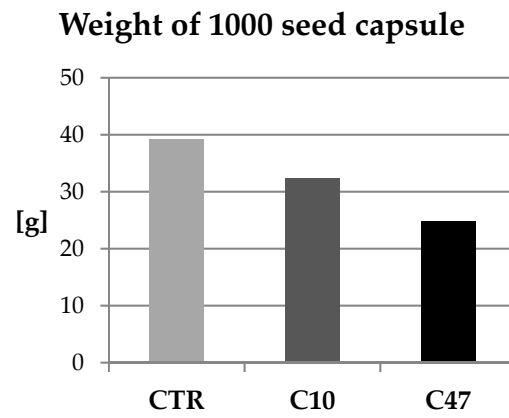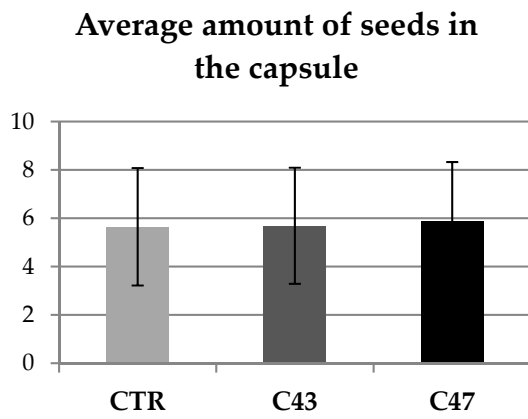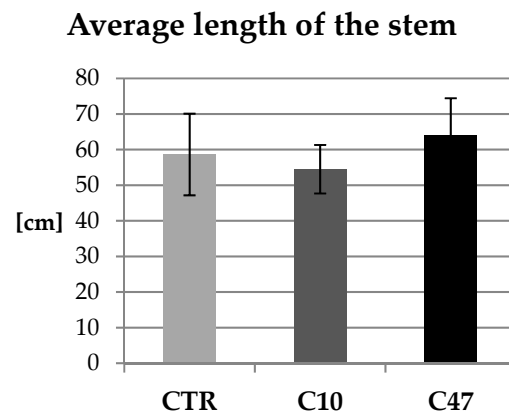

**Figure S2**

Supplement: Supplementary file 1 [file metabolites-13-00437-s001.zip › Figure S2.pdf]

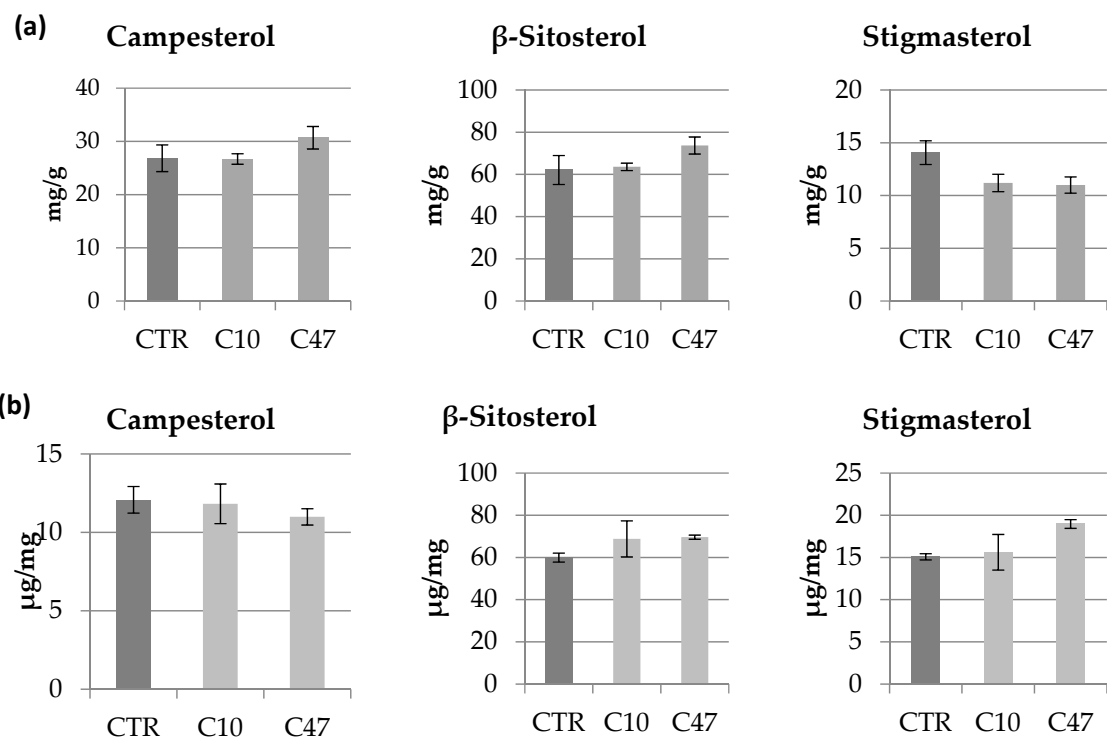

Figure S3

Supplement: Supplementary file 1 [file metabolites-13-00437-s001.zip › Figure S3.pdf]

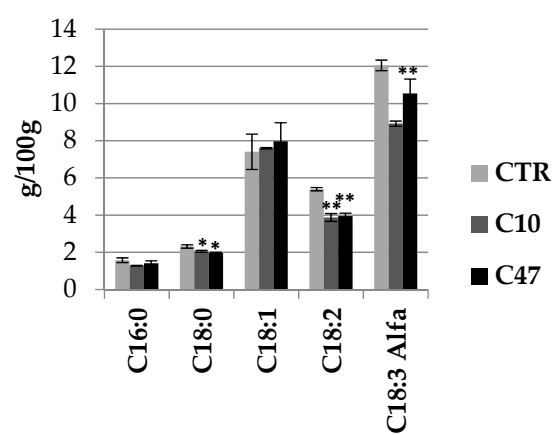

Figure S4

Supplement: Supplementary file 1 [file metabolites-13-00437-s001.zip › Figure S4.pdf]

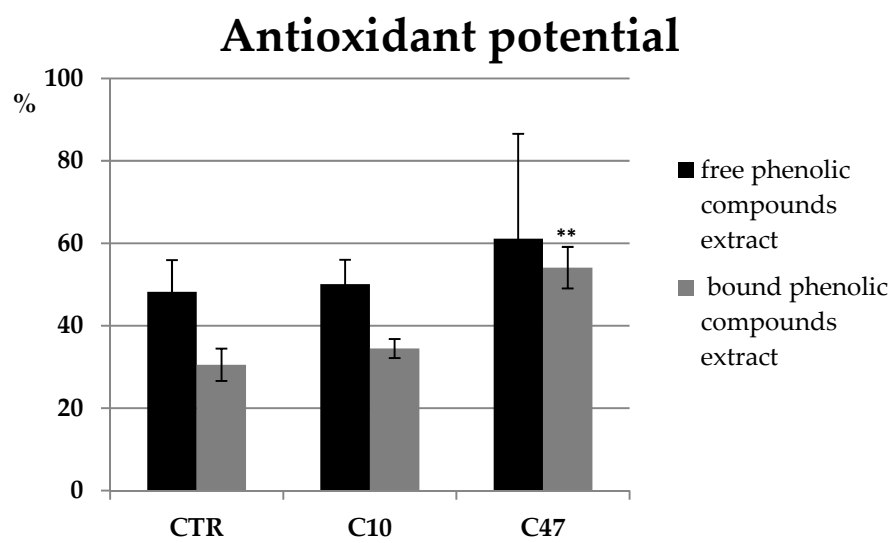

Figure S5

Supplement: Supplementary file 1 [file metabolites-13-00437-s001.zip › Figure S5.pdf]
